# Achieving Human Resource Management Sustainability in Universities

**DOI:** 10.3390/ijerph19020928

**Published:** 2022-01-14

**Authors:** Muhammad Mohiuddin, Elahe Hosseini, Sedigheh Bagheri Faradonbeh, Mehdi Sabokro

**Affiliations:** 1 Department of Management, Laval University, Quebec, QC G1V 0A6, Canada; 2 Department of Business Administration, Faculty of Economics, Management & Accounting, Yazd University, Yazd 8915818411, Iran; elahe.hosseini@stu.yazd.ac.ir (E.H.); bagheri.sedighe@stu.yazd.ac.ir (S.B.F.)

**Keywords:** sustainability management, human resource management sustainability, university, competitive advantage

## Abstract

The sustainability of human resource management (HRM) is the basis for an organization’s future growth and success. This study aims to investigate achieving HRM sustainability in universities. We use a quantitative research method design to investigate the factors that affect HRM sustainability at universities. The study was conducted during the spring and summer of 2020 at Iranian state universities. As the study’s statistical population included 2543 employees, a sample size of 334 employees was calculated using the Cochran formula. A questionnaire with 32 statements based on a 5-point Likert scale was used to collect the data, which were analyzed using PLS3 software. The findings show that human resource practices, social factors, psychological factors, employer branding, and economic factors have positive and significant effects on HRM sustainability at universities. Findings indicate that it is essential to consider the implementation of adequate HRM practices and related socio-economic and psychological supports for HRM sustainability in universities that can lead to the competitiveness of the higher education institutions such as universities.

## 1. Introduction

Human resources (HR) are regarded as the central pillar of an organization’s competitive advantage [1]. Furthermore, because of its importance in optimizing costs and improving productivity and quality, HR is recognized as an essential resource for organizations [2]. Human resource management (HRM) should form functional groups to facilitate collaboration and coordination between an organization’s different components. New approaches emphasize that HR capabilities are fundamental for an organization’s improvement and sustainability [3]. According to researchers, organizational sustainability refers to obviating the stakeholders’ direct or indirect demands while taking care of future stakeholders’ demands. They also believed that the sustainability concept has developed since the 1990s. However, researchers have not comprehensively identified its capabilities in HRM [4]. Sustainability plays a significant role in determining theoretical and pragmatic human resources relationships and developing new perspectives through a “triple bottom line”, including ecological, economic, and social aspects [5].

Enhert et al. [6] argued that it is unwise to disregard HRM as the social aspect of sustainability. They believed that HRM sustainability is crucial for organizations because of increased occupational health issues, shortage of human resources, and an aging population. Based on the HRM definition proposed by the WCED [7] and the challenging task of defining this concept, scholars have identified the following justifications for HRM sustainability: efficiency-oriented, context-bound, and substance-oriented [4,8]. Accordingly, the sustainability of HRM can be distinguished in three different conceptual approaches: sustainable resource management, sustainable work systems, and sustainable HRM. Sustainable resource management aims to clarify the association between an organization and the environment and to propose approaches to deal with the scarcity of resources. Work systems highlight the social aspect of sustainability and intend to expand the perception of mechanisms that result in the implementation and improvement of human resources. Finally, sustainability is regarded as a shared advantage for stakeholders leading to enduring economic sustainability.

Hence, HR sustainability is a set of skills, motivation, values, and trust created to avoid detrimental environmental effects by adopting justice, development, and welfare [9]. Sustainable HRM practices make it possible to achieve the financial, social, and environmental objectives of an organization’s internal and external stakeholders. Moreover, sustainable practices can limit unintended consequences and adverse feedback [8] and seek outcomes that meet stakeholder expectations. These results may be more critical for some organizations than others [10]. Sustainable HRM contributes to developing an influential organizational culture, job security, health promotion, flexibility, participative leadership, sustainable competitive advantage, a value-added economy, self-responsibility, and work-life balance [4]. The approach conceptualizes sustainability as mutual benefit for all stakeholders; moreover, sustainable HRM is interpreted as a cross-functional task. Given that people’s motivation should be regarded as the core of the workplace in organizations, this concept refers to the transition of focus from human control toward resource management [11]. Traditionally, scholars believed that sustainability was associated with HRM in crises, because of lack of skill or employees’ perception of the negative impact of HRM [4].

These days, most organizations are faced with the lack of encouraged and expert employees. Therefore, it is essential to examine the concept of HRM sustainability based on the “triple-bottom-line” approach [12]. Consequently, sustainable HRM may lead to employees’ constant and positive influence on the community along with active participation at the workplace [13]. Hence, this study can contribute to the literature on HRM from two different perspectives: (1) it leads to sustainability influencing employees’ thoughts and behaviors; (2) it can result in enduring welfare of employees physically, economically, and socially when implemented in HRM systems. It is also noteworthy that sustainable HRM demonstrates the social and economic conditions of the organization leading to sustainable development. Higher education institutions (HEIs) are required to implement a holistic perspective on their activities because sustainable development seeks to incorporate social, environmental, and economic issues. Therefore, HEIs should be pursuing a systemic and integrated approach to apply sustainable development into education, regardless of the role of administrative policies. According to [14], the concept of sustainability can be implemented in HEIs within a wide range from the institution level to the state [15]. The present study can also highlight the significance of sustainable development for HEIs because it will be capable of implementing alteration and evolution.

Although sustainability in HEIs has been studied recently, to the best of our knowledge, HRM sustainability in universities has yet to be addressed adequately [16]. Disterheft et al. [17] believed that the academic community has witnessed improvements in the implementation of sustainability, yet it is necessary to move away from unsustainability toward sustainability in higher education. Moreover, HEIs such as universities have failed to pursue a comprehensive paradigm shift from traditional academic structures to the application of sustainability and deal with its potential challenges [18]. Disterheft et al. [17] also argued that the concept of sustainability has been limited to its environmental dimension due to the extensive investigation of the terms SD and going green. Hence, the study of sustainability might be at risk of losing reliability and prominence.

Sustainable HRM is important for universities that compete globally, and examining the factors that affect sustainable HRM for university staff is regarded as the main challenge [19]. Moreover, meeting the current generation’s needs without endangering the abilities and potential of future generations has been considered essential for universities [20]. Universities are specialized and professional organizations that offer knowledge-intensive services within a complex organizational structure, and human resources are the main ingredient for producing these services. Hence, universities are a source of changing structures, creating novel and sustainable functions, and institutionalizing these functions within structures toward sustainability. In other words, the designers of educational institutions can change their structures so as to optimize energy consumption, water resources, as well as heating and cooling systems. HEIs can also demonstrate new and sustainable performances in the manufacturing, services, and distribution sectors by establishing research and education units and communicating with governmental companies, industries, and groups. Universities, as private and non-profit institutions, can be responsible for solving community problems [21].

On the other hand, universities have internal goals such as knowledge creation, skills development, motivation, and trust-building. In addition, universities have some extra-organizational goals such as creating an active and successful workforce for society to fulfill the purposes of welfare, health, commitment, justice, long-term development, and creating a welfare society. HEIs play a significant role in achieving sustainability because universities spread sustainability principles through education, research, and communication with stakeholders. Their principal goal is to increase and strengthen awareness, knowledge, ability, and values to build a sustainable and fair system based on educational and academic freedom, where new ideas are nurtured, and up-to-date solutions are sought to create a sustainable lifestyle.

Since there is a shortage of research on HRM sustainability and its dimensions in universities, this study aimed to investigate this research area to fill the theoretical gap in the literature. The major contribution of this study to the existing literature is the examination of HEIs’ perspectives on sustainable HRM. The findings of this study recommend that HEIs should be aware of the significance of this issue. It is also noteworthy that environmental and organizational factors can lead to HRM sustainability in universities. Since it is a relatively unexplored area of research, this study intends to investigate the relationship between these factors based on data collected from three state universities in Iran (Shiraz, Yazd, and Isfahan Universities). Consequently, investigation of sustainable HRM from the HEIs’ perspective is regarded as the novelty of this study.

## 2. Theoretical Framework and Hypotheses Development

Sustainability is an essential principle for HRM and contributes to achieving organizational goals and performance, which reduces harmful effects on stakeholders and maximizes organizational outcomes [22]. It also relates to preserving and enhancing the well-being of present and future generations, ensuring continuity of cultural values, and creating a balanced living environment to enhance the quality of life [23]. It may help eliminate social gaps and inequalities. Thus, HRM developers play an essential role in optimizing costs and improving productivity and quality [10].

HR and HR strategies have been identified as essential factors for organizational success [24]. HR sustainability refers to a set of activities that organizations must perform to have sustainable access to trained workforce, where the aims are their ethical obligations and responsibilities to employees or society [25]. Sustainable strategies emphasize the determining role of HR [10], where there is a difference between strategic HRM and HR sustainability. Strategic HRM primarily focuses on financial outcomes and an organization’s workforce, implementing HRM practices, and monitoring human capital [26]. Kramar [27] states that managers should think beyond the strategic level to create an innovative work environment with social participation from inside and outside. They should also increase responsiveness and responsibility for environmental protection and improve resource allocation and utilization to enhance organizational accomplishments in a competitive environment. Sustainability’s ultimate goal is motivating the organization to achieve economic, social, and environmental performance [10].

HR strategies must satisfy both employee interests and stakeholder’s needs. The interactive approach incorporates a broader range of values and activities related to collective concerns [27] and explains the convergence of procedures between HR sustainability and organizational sustainability. Furthermore, previous studies on HR sustainability have similarly highlighted the human and social aspects are both important for organizational sustainability. These studies accentuate two separate and complementary approaches to human resources: organizational sustainability and sustainable HRM practices [28]. The former focuses on the role of HR in supporting sustainable activities [29], while the latter highlights adopting measures that influence individuals and groups to develop perspectives and behaviors that are consistent with a sustainable approach [30]. Additionally, most universities have attempted to raise their environmental awareness through education. Due to increasing social concerns about environmental destruction and the increasing demand for movement and change toward a sustainable society, universities worldwide have changed their goals and methods. They have included sustainability in every aspect of their organizations [31]. Given the role of universities in enhancing society through educating and affecting the youth, the present study aimed to determine the underdeveloped aspects of sustainability implementation in universities. Therefore, this study’s fundamental hypothesis is that:

**Hypothesis** **0** **(H0).** *Various Factors Have Different Impacts on HRM Sustainability at Universities.*

Organizational factors are among the most fundamental variables that affect HRM sustainability. According to the International Standardization Organization’s (ISO) model, organizational sustainability identifies opportunities, changes, and trends in the external environment ISO/CD9004 [32], promoting jobs and profitability while considering society and the environment. It provides solutions that improve people’s lives and meet basic needs with the least environmental impact and highest social performance for the present and future [33]. It also helps HRM develop the mentality and actions that lead to organizational sustainability [34]. Some practices, such as talent acquisition, training, knowledge acquisition, and skill and ability development programs, contribute to organizational HR sustainability objectives [10]. Organizational sustainability should be grounded on validating, recognizing, and enhancing the capabilities of professionals. If these issues are not addressed, the organization will have a strong tendency to lose talent [13].

Moreover, HRM can contribute to organizational sustainability in the following five aspects [11]: organizational change (value and behavior); workplace systems and institutions (recruitment and awards); professional development and organizational training (education and raising capabilities); employee participation and consultation (innovation); and work-life balance. It will be essential for organizations to integrate the sustainability of environmental and individual factors. Developing human capabilities through effective HRM systems, implementing teamwork approaches, and continuing education may be crucial in environmentally friendly measures [11]. Sustainability perspectives can lead to the creation and survival of successful organizations. Hence, there is a relationship between HRM and sustainable organizational development. Therefore, it can be argued that HR is a factor in organizational success [35]. HRM is also a process that supports organizational activities in dealing with various problems and helps achieve predetermined goals [36].

Manager cooperation, as a dimension of HR, plays a crucial role in an organization’s employee sustainability and job satisfaction through employee performance appraisals [37]. Therefore, developing sustainable HRM approaches is associated with reducing complexity, placing appropriate emphasis on appropriate relationships regarding stakeholder demands, taking proper measures to improve organizational resources, developing recruitment processes, paying viable salaries and rewards, highlighting learning and actual HR development, and implementing a performance management system, as well as preserving the organization’s HR. Consequently, the concept of sustainable HR is regarded as an inseparable part of an organization’s macro strategy [38]. Moreover, universities should seek sustainable development so that they can enhance sustainable behaviors among employees and students as well as moderate and decrease the operating effects on the environment [39,40]. This discussion leads to the following hypothesis:

**Hypothesis** **1** **(H1).** *HR practices have a significant effect on HRM sustainability.*

Social sustainability and occupational changes are crucial processes that affect the economy [41]. Social factors include organizational social responsibility and other significant factors, such as the social infrastructure, availability of job opportunities, meeting psychological conditions, social justice, and social sustainability [1].

Job opportunities are crucial for social sustainability. Beyond providing income, employment allows people to experience social well-being through interactions with others [42]. The design of social sustainability involves education, public services, a green atmosphere, values and vacations, affordable accommodation, environmental quality, and sustainable municipal scheme that have direct or indirect impacts on the social environment [43]. Finally, social sustainability includes a set of ideas such as justice and the quality of work-life balance, which influence spiritual and moral standards [44]. It is defined as having access to essential services and goods, security, and a sense of belonging, empowerment, solidarity, and happiness [45]. This discussion suggests the following hypothesis:

**Hypothesis** **2** **(H2).** *Social factors have a significant effect on HRM sustainability.*

Psychological factors, including anxiety, job attitude, and job-related stress, can cause changes in well-being [46] (Mazur, 2014). Safety and a sense of social identity also belong to psychological needs [42]. This is one of the critical and essential factors in the stability of psychological work-related stress relief. These capabilities are related to efficiency, which can play a crucial role in employee sustainability [47], improve the quality of life, and expand the sustainability paradigm from the environmental to the psychological and social context [48]. The core of psychological sustainability is associated with positive organizational behavior.

Employee stressors are mainly associated with detrimental work behaviors. Adverse effects of cultural dimensions (individualism and collectivism) and dealing with work-related stress have been observed in psychological pressure or stress [13]. Therefore, organizations should consider the relationship between individual and organizational health. A healthy organization can achieve its social and business objectives; moreover, if employees can relate their identity to the organizational objectives, they are more likely to be committed [19]. Hence, such objectives will be recognized as a source of satisfaction and individual development [41]. Based on this discussion, we propose the following hypothesis:

**Hypothesis** **3** **(H3).** *Psychological factors have a significant effect on HRM sustainability.*

Employer branding refers to a set of efforts for communicating with employees and establishing the organization as an excellent place to work. As a competitive advantage, power can promote growth and development [49]. A brand value is formed by utterly satisfying stakeholder expectations, which include an excellent organizational reputation [50]. Moreover, a good reputation can lead to a sustainable competitive advantage in terms of the perceptions of potential employees in the HR market [51]. Brand fairness refers to the outcome of previous investments in a marketing synthesis, which adds value to services [52].

Qualified faculty members and employees can generate outstanding intellectual capital that influences the organization’s performance and other outcomes to survive in this competitive environment [53]. There is a global shortage of talent and expertise, and many researchers have developed strategies for attracting talented applicants. However, most of these are short-term strategies designed for new job positions in an organization [54]. When most of a university’s value (around 70%) lies in its intangible assets, is known as an attractive employer is regarded as a competitive advantage [55]. It is believed that employees’ enduring commitment and inclination to the values of organizational brand are necessary while seeking the shift toward sustainability. According to Srivastava et al. [56], this transformation is challenging, complicated, durable, and burdensome. Although the main objective of universities might be to educate and perform administrative functions, teaching and research are less likely to highlight sustainability. Bice and Coates [57] argued that such inconsistencies may result in declined awareness and value to university brand image among employees. Besides, newly recruited staff and employees in some other universities may not be fully committed to the university brand value. Therefore, the brand image and sustainability will be negatively affected, which necessitates the need for a transformation of the paradigm within different dimensions of universities [58].

This discussion leads to the following hypothesis:

**Hypothesis** **4** **(H4).** *Employer branding has a significant effect on HRM sustainability.*

Sustainable development refers to the long-term and systematic exploitation of natural resources, given that we should also preserve these resources for future generations. Such development enables countries to experience economic and social improvements without destroying their environmental resources [6]. Organizations should also consider a collection of processes, strategies, and economic factors for systematic and continuous environmental management [59]. Economic sustainability is achieved through better resource management, process productivity, cost reduction, and economic management [60] (Cuccia, 2015). Hence, economic sustainability, facility growth, macroeconomic policies, and job guarantees are fundamental economic factors for enhancing management and the competitive advantage of human capital, HR performance, reengineering, cost reduction strategies, and senior management engagement [1].

In classical endogenous growth theory, human capital plays a central role in technological advancement and sustainable economic growth [61] (Xu and Li, 2020). Social security increases labor productivity and provides the power to develop economic sustainability through accumulating human capital [62] (Martin et al., 2005). Economic sustainability means choosing an option that, based on the best available economic knowledge, leads to overall economic growth and long-term development [63]. The above discussion suggests the following hypothesis:

**Hypothesis** **5** **(H5).** *Economic factors have a significant effect on HRM sustainability.*

Organizational workplace politics are an inevitable fact, and failing to deal with them realistically will predictably be detrimental to the organization [64]. Workplace politics involve encouraging management to achieve unnecessary goals through informal means [65]. Political factors concern clarifying the normative dimensions, a significant expectation from science in developing political stability that requires researcher commitment and institutional support [66]. Employee political behaviors include a wide range of effective tactics, such as improvements in organizational position, reform, and maintenance [67]. The political perceptions of an organization’s employees are based on intentional actions using power, strategies, and legal authority to influence behaviors. Ethical organizations will be successful [68]. According to Cebrián and Junyent [69], if leaders insist on implementing any policies that only focus on the outcome and not the employees, there will be a decrease in employees’ willingness and obligation to seek organizational objectives.

**Hypothesis** **6** **(H6).** *Workplace politics have a significant effect on HRM sustainability.*

**Figure 1:**
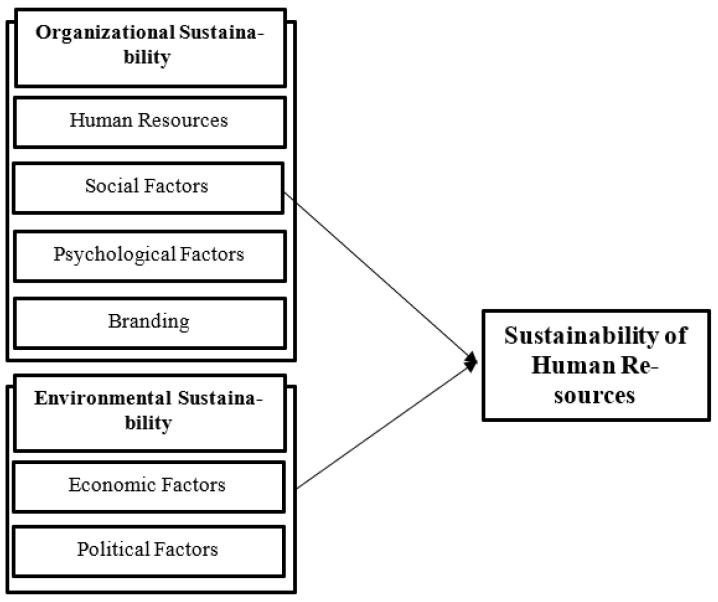
Conceptual model (Source: Authors’ elaboration).

Finally, HRM sustainability can be described as the most complex challenge in the HR field. HRM theories, models, systems, and processes have shifted from a profit-driven motivation to a simultaneous three-way objective called the triple bottom line (TBL), which includes expanding social, environmental, and economic outcomes and addresses social, environmental, and economic issues [70]. HRM sustainability process occurs through developing employee skills and maintaining a healthy and active workforce based on a theoretical process [34]. In addition, Dubey et al. [71] designed a model in which complex dimensions (strategy, technology) and policy and soft dimensions (human resources) are integrated into green supply chain management. Tooranloo et al. [1] studied the influential factors for the successful implementation of HRM sustainability and emphasized that HRM sustainability helps organizations achieve sustainability. However, researchers have conducted various studies in this field and have concluded that insufficient research has addressed the causes of HRM sustainability at HEIs [11]. The authors of previous studies include four factors of organizational sustainability: HR, social factors, psychological factors, and the employer’s brand. They also include economic and political factors as part of environmental sustainability [44,61,65]. Finally, the research’s conceptual framework is presented in Figure 1 as follows:

## 3. Research Methodology

This descriptive-correlational study is applied in terms of its developmental purpose and quantitative data collection. The purpose of applied studies is to develop applied knowledge in a specific field. The study’s statistical population included 2543 employees of three state universities in Iran, namely Shiraz, Yazd, and Isfahan Universities. The managers of the sample organizations declared the necessity of HR sustainability to avoid destruction, reduce electricity intake, and protect the environment. In addition, these universities achieved high ranks based on Greenmetric World University Ranking (UI Greenmetric World University Ranking) in 2020 and 2021. It was also easier for authors to access these three universities because of their locations, particularly during the COVID-19 pandemic. As structural equation modeling was used for the analysis, the sample size should be 5 to 10 times the number of questionnaire items. Therefore, based on Cochran’s formula, 334 employees were selected. Based on the analysis of the quantitative data, 73% of the respondents were male, and 27% were female; besides this, 78% held a Ph.D. degree, and 22% held a Master’s degree. Moreover, 23% of the participants were single, and 77% were married. Finally, 17% of the respondents had up to 5 years of experience, 63% had between 5 and 10 years of experience, and 20% had over 10 years of experience. Since there were no standard questionnaires on this topic, a researcher-made questionnaire (Appendix A) was developed after reviewing related literature. The proposed questionnaire included 32 questions measured using a 5-point Likert scale (1—Strongly disagree, 2—Disagree, 3—I have no opinion, 4—Agree, and 5—Strongly agree). Likert scale is a tool for measuring people’s attitudes and is used to prepare attitude questionnaires in management and the humanities. In general, three standard scales have been introduced by Rennes Likert, known as the five-degree, seven-degree, and nine-degree scales. These scales can be used to express agreement or determine the importance of items. The most common form of the Likert spectrum is 5 degrees. This scale measures only the subject and issue under study and not another irrelevant issue. It also expresses a more or less positive or negative tendency and not an indifferent tendency. The main questionnaire items were compiled and distributed in person and online. Due to the ineligibility and repetitiveness of some responses, a total of 297 questionnaires were collected and analyzed.

Moreover, informed consent forms were distributed among these employees to indicate their voluntary participation in the project, and they were assured that the collected data would only be used in this study. They were also free to withdraw from the project at any time without any adverse consequences. In this study, HRM sustainability is considered the dependent variable, and organizational and environmental sustainability are considered the independent variables. Smart PLS3 software, which does not require a normal distribution, was used to analyze the data [72]. It is noteworthy that the structural model is more significant than the measurement model. Moreover, the path model presented in this method can be applicable to small samples. Initially, to confirm the face and content validity of the questionnaire, the judgment and approval of experts were taken into account. We also identified the relevance, simplicity, and clarity of each item through a review of the subject literature and the experts’ feedback. Then, the structural equation modeling of convergent and divergent validity was used to measure construct validity. The reliability of the questionnaire was measured using Cronbach’s alpha and combined reliability. The results shown in Table 1 indicate that the research instrument has excellent validity and reliability. AVE was measured to determine convergent validity; the values obtained are all higher than 0.5, which indicates strong validity. The results also show that Cronbach’s alpha and the joint reliability values are beyond the appropriate minimum of 0.7. These test results all indicate good validity and reliability.

The mean-variance index was extracted to determine convergent validity, and the root means index of the extracted variance was used to check for divergent validity. The obtained root mean values of the extracted variance are higher than 0.5, which indicates that the variables have divergent validity.

Based on the results obtained using the Smart PLS 3 software (SmartPLS GmbH; Bönningstedt; Germany, and presented in Table 1 and Table 2, validity (convergent and divergent) and reliability (reliability, combined reliability coefficient, and Cronbach’s alpha) are acceptable.

## 4. Results

**Figure 2:**
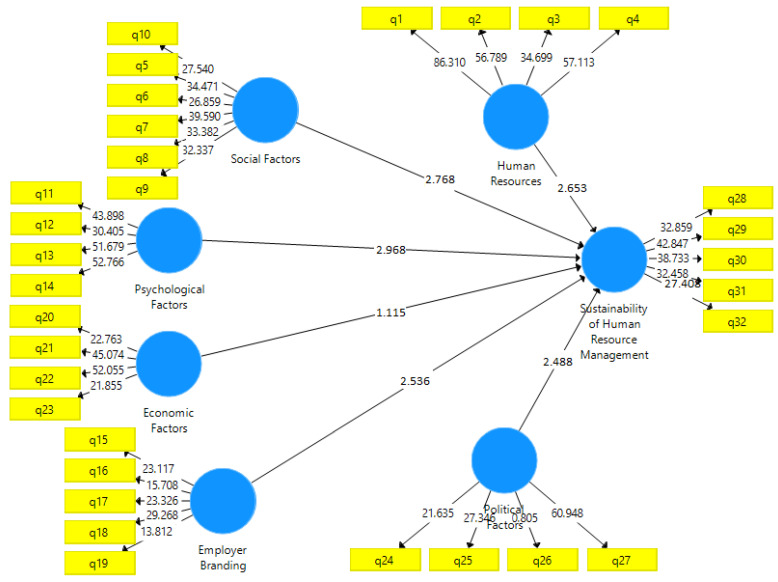
*t*-values (Source: authors).

To investigate the structural model’s fitness, assessments were conducted at the measurement, structural, and general levels. Several criteria were employed to assess model fitness using the partial least squares method, such as the coefficients of the t-statistic significance values. The significance of such coefficients can be confirmed at the 95% confidence level if they are higher than 1.96. The results of this criterion confirmed the significance of coefficients whose critical values are higher than 1.96 (Figure 2).

The findings indicated a significantly positive relationship between the components of human resource practices and the sustainability of human resource management. According to the obtained t-statistics value for this relationship (2.653) the first hypothesis was confirmed. The second hypothesis, reflecting the positive and significant effect of the social components on the sustainability of HRM, was confirmed as the obtained t-statistics value for this relationship was 2.768. The third hypothesis refers to the significantly positive effect of the psychological components on HRM sustainability. It was approved because the obtained t-statistics value for this relationship was 2.968. The fourth hypothesis refers to the significantly positive effect of employer brand on the sustainability of human resource management, which was confirmed because of the obtained t-statistics value of 2.536. The fifth hypothesis, highlighting the effect of economic components on the sustainability of human resource management, was however rejected since the obtained t-statistics value (1.115) is lower than the critical value (1.96) at the 95% confidence level. The sixth hypothesis concerning the direct effect of political components on the sustainability of human resource management was approved because the obtained t-statistics value was 2.488.

### 4.1. Coefficient of Determination (R^2^)

**Figure 3:**
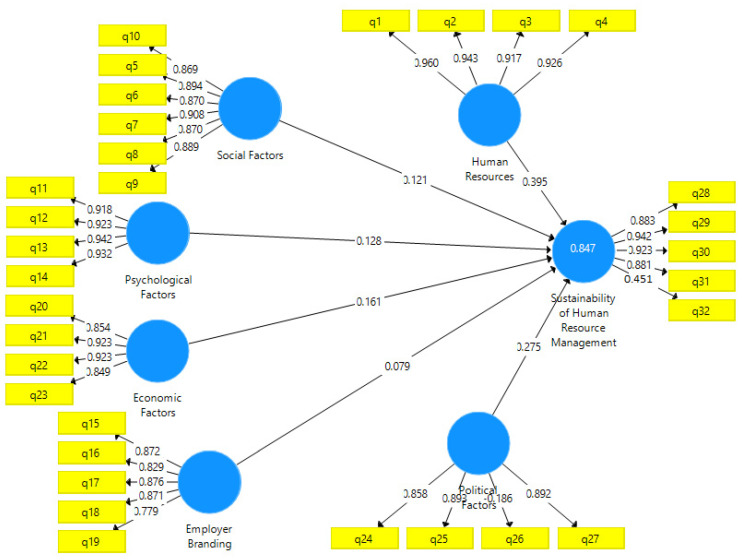
R^2^ (Source: authors).

The R^2^ coefficients was used to examine the intensity of relationships between dependent structures. R^2^ is regarded as a measure explaining the exogenous variables’ influence on an endogenous factor; accordingly, 0.19, 0.33, and 0.67 indicate weak, medium, and robust R^2^ values, respectively. In this study, the criterion for HRM sustainability was 0.847, so the structural model showed a good fit at the robust level. The results are shown in Figure 3.

The results of Figure 3 also revealed that the impact factor for human resources on the sustainability of human resource management is equal to 0.395, the impact factor of social factors on the sustainability of HRM equals to 0.121, the obtained impact factor of psychological factors on HRM sustainability is 0.128, the impact factor of the employer brand on the sustainability of HRM is equal to 0.079, the obtained impact factor of economic factors on the sustainability of HRM is 0.161, and the impact factor of political factors on HRM sustainability is equal to 0.275. Figure 3 shows that the highest impact factor belonged to the human resources variable.

### 4.2. Predictive Relevance (Q^2^)

The Q^2^ criterion proposed by [73,74], determines the predictive power of the independent variables. They believed that models with an acceptable structural fit ought to predict the characteristics of the model’s endogenous structures; that is, if the relationships between structures are appropriately defined, the structures will have a sufficient impact on each other, which will verify the hypotheses. The values for low, medium, and robust predictive power are regarded as 0.2, 0.15, and 0.35, respectively, for all endogenous structures [72]. In this study, the respective value for HRM sustainability was 0.785, indicating that the model has robust predictive power.

### 4.3. Goodness of Fit (GOF)

The overall model contains all calculations and measures of the structural model. Hence, the overall fitness of the model can be confirmed based on the GOF criterion using 0.01, 0.25, and 0.36 to reflect poor, medium, and robust GOF values. The GOF value for the model is 0.897, indicating that the overall fit of the research model is robust.

GOF = √average (Commonality) × average (R^2^)

The standardized root means square residual (SRMR) was implemented as another approximate model fit criterion. The values of 0.05, 0.08, and an SRMR value lower than 0.10 indicate an acceptable fit for the overall model as proposed by [75,76,77] respectively. As shown in Table 3, this index is at an acceptable threshold, supporting the fitness of the current research model. The normed fitness index (NFI) has also been used to measure fit. The potential range of this index is between 0 and 1, but the values greater than 0.9 are considered acceptable [72]. Table 3 reports that this value is 0.918, again indicating supporting the model’s fitness.

Finally, t-statistics were employed to examine hypothetical relationships amongst variables. Six sub-hypotheses were developed to assess the primary hypothesis. As shown in Table 4, the t-coefficient for the five existing relationships has been verified. The standardized factor loading coefficients associated with the paths of each hypothesis were analyzed to assess the influence of predictor variables on dependent variables. Such values suggest that changes in independent variables might partially contribute to shifts in the dependent variables.

## 5. Discussion

This research was designed to explore the factors affecting HRM sustainability in universities. The current literature shows that much attention has been paid to sustainability during the past two decades, which has led to an increase in sustainability-related measures. Universities are influential in societies; they play a central role in developing science, technology, innovation, justice-orientation, talent development, empowerment, and wealth-creation while strengthening the spirit of cooperation and responsibility and fostering an environment for thinkers, researchers, and professionals. They also enhance and disseminate new ideas to promoting individual and social life in a suitable environment for free intellectual thought and scientific critique. Therefore, HRM sustainability in universities plays a vital role in achieving those noble goals.

The findings regarding the first hypothesis show that components of HR practices had a significantly positive impact on HRM sustainability. The respective findings are consistent with results of other studies by [34,37], indicating that HRM sustainability is achieved by developing employees’ skills and maintaining a healthy and active workforce. Human aspects and the transition agents’ measures can play significant roles in dealing with the complicated procedure of the shift to sustainability in every community, particularly HEIs [14]. It is also based on a theoretical process highlighting that acquiring knowledge helps achieve sustainability objectives. Additionally, training an organization’s human resources is essential for sustainable development. Employees can significantly affect the progress of university positions in different aspects, including academic reputation, high-quality academic plans, and research quality [78]. Thus, Aust et al. [79] asserted that HRM measures can trigger employees’ performance, which will lead to the enhancement of university performance.

Provided that the staff acts with satisfaction and determination to develop the organization, university empowerment is developed through strategic development planning, supporting research and innovation, and developing future activities, research, and professional skills. Increasing employees’ independence and motivation enhances learning and creativity opportunities at the workplace. Furthermore, the university staff’s responsibilities and ethical obligations have led to adherence to principles and regulations. Sustainable talent development is achieved through the direct experience of acquiring knowledge and skills. Unique mechanisms such as succession planning, motivation techniques, teamwork, training, coaching, specialized seminars, self-development, knowledge sharing, hands-on learning, and networking can enhance talent and ultimately create HRM sustainability at the university level.

The second hypothesis refers to the positive and significant impact of social components on HRM sustainability. This result is consistent with research by [1,15,23,43], argued that HEIs should take into account the behaviors and insights of the stakeholders so as to experience a better understanding of their priorities and demands. Consequently, they will be able to set objectives and strategies, observe these strategies for accountability, and develop community–university involvement. According to Hamón et al. [80], this procedure will result in a highly sustainable university. Similarly, Lozano et al. [81] asserted that employees should be granted comprehensive sustainability proficiency in order to improve the future generation’s perspective. Accordingly, social sustainability design involves education, public amenities, green space, recreation, available accommodation, environmental quality, and sustainable urban design, which can have direct or indirect impacts on the social environment. Social sustainability also helps create and improve the quality of life, eliminates social gaps and inequalities, and connects the cultural values of the past, present, and future by ensuring continuity. Social justice should be observed in universities because this factor causes employees to accept the organizational structure, respect organizational rules, and make sacrifices for the interests of the organization and protection of organizational resources. This factor urges employees to be actively involved in managing the organization’s affairs and fulfilling their social responsibilities at the university. Universities can also increase their practical cooperation with students and professors from different cultures and establish relationships with universities at the international level to expand multicultural diversity in developing HRM sustainability.

The third hypothesis refers to the significantly positive effect of the psychological components on HRM sustainability. This result is consistent with previous studies by [47], highlighting the core of psychological stability combined with the organization’s positive behavior as a factor in improving the quality of life. Similarly, Moreira et al. [82] believe that engagement theorists have defined disengagement as the conceptual counterpart of engagement, which is an unintentional expression of psychological processes. Consequently, disgruntled thoughts (mental withdrawal and lack of attention), lack of behavioral effort or resilience, and disaffected emotions such as apprehension, grief, and boredom are the indicators of disengagement. They consider freedom from work-related stress an essential part of sustainability and aim to extend the sustainability paradigm from the environmental sphere to the psychological and social context. Since this factor increases and influences teamwork and communication, job stress should be managed, controlled, and significantly reduced by identifying resources and increasing productivity. Efforts should also be made to meet the psychological needs of university staff, including human qualities, organizational beliefs, self-reconstruction and self-esteem, self-awareness, and a sense of belonging.

Meeting employees’ psychological needs provides the basis for survival, well-being, and workplace advancement, where the work environment is compatible with individuals’ abilities and capabilities. This can be done by holding in-service training sessions, workshops, and similar activities that increase creativity within the organization, which aids in development and advancement while strengthening and influencing job growth opportunities. Furthermore, these factors can affect HRM sustainability and lead to growth, development, and achievement of academic goals.

The fourth hypothesis refers to the significantly positive effect of employer brand on HRM sustainability. Thus, the collected data support this hypothesis. These findings are consistent with [49], who state that brand stability is effective in changing power relations in the global arena, and superior firm stability is regarded as a competitive advantage that leads to business growth and increased sales. The increasing number of educational programs at universities, raising community awareness concerning environmental issues, and the development of new information technologies require novel capabilities. Therefore, there is need for competition and flexibility among institutions [83]. This study’s findings suggest that top universities can positively affect the creation of an academic reputation, which adds to the realization of all long-term objectives inside and outside the university. In addition, university performance, high-quality education and faculty members, and research and student output are required for excellence in a university environment. A superior university contributes to satisfying future demands while meeting the current needs of the university and community. Managers’ commitment to implementation of sustainability in HEIs can result in employees’ compliance with the brand objectives and the formation of a competitive brand. Therefore, the brand has a significantly positive impact on HRM sustainability.

The fifth hypothesis refers to the effect of economic components on HRM sustainability. This result is inconsistent with the findings of [1]. Authors argued that economic stability is achieved through better resource management, process efficiency, cost reduction, and economic management. Sustainability highlights the prominence of protecting the ecosystem and establishing the implementation of sustainability measures in public and private institutions [84]. Unlike the disapproving economic statistics and fluctuation in the economic conditions in Iran, there has been a growth in smart city projects to achieve sustainability objectives. Nonetheless, the severe sanctions against the Iranian government have had a detrimental impact on human resource sustainability. In addition, increased working hours, which are imposed by high inflation rates and the devaluation of national currency, have declined the efficiency and productivity of institutions such as universities. Consequently, it is recommended to assess universities based on the environmental conditions, their employees’ performance, their capabilities and expertise, and the degree of occupational complicatedness. An efficient system of rewards and equitable distribution and non-discrimination in paying rewards can play a vital role in increasing employees’ motivation, commitment, satisfaction, and performance. Commitment by the university’s senior management to economic sustainability, macroeconomic policies, and efficiency can be positive steps toward reducing the economics of HR. If such steps are carried out within the framework of university policies with the cooperation of relevant units, and all aspects of HRM sustainability are observed, they will have a positive effect and lead to growth and development.

The sixth hypothesis refers to the direct effect of political components on HRM sustainability. This result corroborates those of [65,67], who stated that clarifying normative dimensions and political stability are necessary; this is one of the inseparable components of the research. According to Argento et al. [85], HEIs are forced to comply with the governmental demands to be more sensitive towards sustainability. In addition, Ekman et al. [86] argued that if some academics who are more sensitive towards sustainability intend to operationalize some norms and values into their institution, it may lead to normative isomorphic pressures due to the unremitting dialogue with their colleagues and program directors. There are significant expectations from science regarding sustainable development because it requires both researcher commitment and institutional support. Therefore, universities should act based on up-to-date scientific standards through policies and performance standards and by considering the employees of the educational departments as the university’s crucial staff. Universities should provide the staff and faculty members with the necessary equipment and programs and properly implement them to achieve scientific progress. Universities should adopt institutional policies that may lead to the gaining of legitimacy among other universities. Moreover, by adopting a variety of policies and procedures, long-term goals inside and outside the university can be realized while meeting the current needs of the university and society. This helps the university meet future needs, control unwanted consequences, and avoid negative feedback to sustain HRM.

## 6. Conclusions

According to the results of this study, HRM sustainability at HEIs is a relatively new paradigm. It should be investigated along with other valuable and practical components of environmental, socio-cultural, and human resource practices, as well as organizational, psychological, economic, and political factors at different levels (i.e., individual, organizational, society). Furthermore, dynamics in temporal dimensions (short-term and long-term) should be highlighted because interactions and relationships between different levels are needed to achieve the goals. Objectives should be achieved using different organizational resources so that society’s existing needs do not endanger the future and control negative feedback. Applying sustainability principles in HRM practices also provides workers with enduring social, economic, and physical well-being. Due to the development of universities, long-term goals inside and outside universities are achieved by creating a balance between work and life.

Given that stakeholders, policymakers, and consumers have realized that certain activities may have adverse environmental effects, HEIs need to consider the sustainability of human resources to ensure their long-term success. In addition to acting as an efficiency measure, sustainable HRM can support self-sustained development processes. Regardless of inadequate evidence in the literature regarding a clear definition of HRM sustainability and the lack of emphasis on this issue, this study shed some light on the meaning of sustainability and its relationship with HRM.

### Limitations and Future Research

Despite all of its contributions, the present study cannot be without limitations. For instance, some of the university employees were reluctant to participate due to their conservative personality or biased responses; hence, the generalizability of the findings of this study are called into question. Moreover, there are different factors and characteristics regarding sustainable HRM in various cultures that make it difficult to generalize the findings to all the societies. Consequently, other researchers are recommended to conduct similar studies in different universities or different cultures using the same model. Furthermore, it is suggested to identify and investigate other intra- and inter-organizational outcomes in sustainable HRM in different universities. Given its dynamic process, sustainability does not refer to rigidness. Accordingly, it is essential to conduct further studies to explore sustainability in HRM.

## Figures and Tables

**Table 1:** Composite reliability, Cronbach’s alpha, AVE (Source: authors).

Row	Construct	Variables	Index	Cronbach’s Alpha	CR	Shared Reliability	AVE	R^2^	Q^2^
1	Organizational Stability	Human Resources	1–4	0.953	0.966	0.955	0.878	-	-
		Social Factors	5–10	0.944	0.955	0.947	0.781	-	-
		Psychological Factors	14–11	0.947	0.962	0.948	0.863	-	-
		Employer Branding	15–19	0.900	0.926	0.902	0.716	-	-
2	Environmental Stability	Economic Factors	20–23	0.910	0.937	0.913	0.788	-	-
		Political Factors	24–27	0.809	0.987	0.909	0.591	-	-
3	Stability of HRM		28–32	0.814	0.927	0.889	0.660	0.847	0.785

**Table 2:** Convergent validity (Source: authors).

Variables	1	2	3	4	5	6	7
Economic Factors	0.888						
Employer Brand	0.810	0.864					
Human Resources	0.824	0.854	0.937				
Political Factors	0.810	0.746	0.699	0.869			
Psychological Factors	0.770	0.840	0.776	0.790	0.929		
Social Factors	0.872	0.859	0.883	0.765	0.805	0.915	
Management Stability of Human Resources	0.847	0.806	0.863	0.815	0.807	0.812	0.869

**Table 3:** SRMR and NFI (Source: authors).

	SRMR	NFI
Acceptable values	0.10≥	0.9≤
Calculated values	0.076	0.918

**Table 4:** Path relationships (Source: authors).

Path	*t*-Test	Influence Coefficient	Result
Human resources have a significant impact on the sustainability of HRM	2.653	0.395	Verified
Social factors have a significant impact on the sustainability of HRM	2.768	0.121	Verified
Psychological factors have a significant impact on the sustainability of HRM	2.968	0.128	Verified
Branding of employer factors have a significant impact on the sustainability of HRM	2.536	0.079	Verified
Economic factors have a significant impact on the sustainability of HRM	1.115	0.161	Denied
Political factors have a significant impact on the sustainability of HRM	2.488	0.275	Verified

## Data Availability

Not applicable.

## Appendix A. Questionnaire

1 = Strongly disagree, 2 = Disagree, 3 = Neither agree nor disagree, 4 = Agree, 5 = Strongly agree.

Organizational Stability

Human Resources

1. To what extent do employees’ independence and motivation affect the development of HRM sustainability in organizations?
2. To what extent does employees’ empowerment affect the development of HRM sustainability in organizations?
3. To what extent does employees’ responsibility affect the development of HRM sustainability in organizations?
4. To what extent does employees’ ethical commitment affect the development of HRM sustainability in organizations?

Social Factors

5. To what extent does multicultural diversity affect the development of HRM sustainability in organizations?
6. To what extent does social equity affect the development of HRM sustainability in organizations?
7. To what extent does social responsibility affect the development of HRM sustainability in organizations?
8. To what extent does availability of job opportunities affect the development of HRM sustainability in organizations?
9. To what extent does workplace creativity affect the development of HRM sustainability in organizations?
10. To what extent do employees’ relationship and collaboration affect the development of HRM sustainability in organizations?

Psychological Factors

11. To what extent does freedom from work-related stress affect the development of HRM sustainability in organizations?
12. To what extent does meeting psychological needs affect the development of HRM sustainability in organizations?
13. To what extent do organizational conflicts affect the development of HRM sustainability?
14. To what extent do active perspectives affect the development of HRM sustainability in organizations?

Employer Branding

15. To what extent does brand attractiveness affect the development of HRM sustainability in organizations?
16. To what extent does organizational reputation affect the development of HRM sustainability?
17. To what extent does job security affect the development of HRM sustainability in organizations?
18. To what extent does brand identity affect the development of HRM sustainability in organizations?
19. To what extent does brand image affect the development of HRM sustainability in organizations?

**Environmental Stability**

Economic Factors

20. To what extent does impartial distribution of income affect the development of HRM sustainability in organizations?
21. To what extent does effective reward system affect the development of HRM sustainability in organizations?
22. To what extent does executive manager’s commitment to economic sustainability affect the development of HRM sustainability in organizations?
23. To what extent do economic macro-policies affect the development of HRM sustainability in organizations?

Political Factors

24. To what extent do performance policies and standards affect the development of HRM sustainability in organizations?
25. To what extent do institutional policies affect the development of HRM sustainability in organizations?
26. To what extent does policy and procedure diversity affect the development of HRM sustainability in organizations?
27. To what extent does ethnic policy-making affect the development of HRM sustainability in organizations?

Stability of HRM

28. To what extent do attracting and maintaining talents affect the development of HRM sustainability in organizations?
29. To what extent does job system literacy affect the development of HRM sustainability in organizations?
30. To what extent does skilled workforce affect the development of HRM sustainability in organizations?
31. To what extent does green employment affect the development of HRM sustainability in organizations?
32. To what extent does participation in green activities affect the development of HRM sustainability in organizations?
